# To shunt or not to shunt a pulmonary adenomatoid cystic malformation after 33 weeks of gestation: a case report

**DOI:** 10.1590/S1516-31802008000400011

**Published:** 2008-07-03

**Authors:** Rodrigo Ruano, Paula Beatriz Tavares Fettback, Vinicius Lima Ribeiro, Marcus Marques Silva, João Gilberto Maksoud, Marcelo Zugaib

**Keywords:** Cystic adenomatoid malformation of lung, congenital, Ultrasonography, Prenatal diagnosis, Fetal therapy, Abnormalities, Malformação adenomatóide cística congênita do pulmão, Ultra-sonografia, Diagnóstico pré-natal, Terapias fetais, Anormalidades

## Abstract

**CONTEXT:**

Macrocystic adenomatoid malformation of the lung can cause severe mediastinal shift, hydrops and polyhydramnios, thereby increasing the risk of perinatal deaths. After 33 weeks of gestation, repeated puncturing of the cyst is recommended. We present a case in which a cyst-amniotic shunt was placed instead of performing this procedure.

**CASE REPORT:**

A cyst-amniotic shunt was placed at 33 weeks of gestation because of a large macrocystic adenomatoid malformation of the lung associated with severe mediastinal shift and polyhydramnios. Although it was confirmed that the catheter was in the correct place, the cyst increased in size again two weeks later, associated with repetition of polyhydramnios. It was postulated that the catheter was blocked, and we chose to place another catheter instead of performing repeated punctures. The cystic volume, polyhydramnios and mediastinal shift regressed progressively. At 38.5 weeks, a 3,310 g male infant was delivered without presenting any respiratory distress. The infant underwent thoracotomy on the 15^th^ day of life. Thus, in the present study, we discuss the possibility of placing a cyst-amniotic shunt instead of performing repeated cystic punctures, even at a gestational age close to full term.

## RESUMO

**CONTEXTO:**

A malformação adenomatóide do pulmão tipo macrocística pode causar compressão mediastinal grave, hidropisia e polihidrâmnio aumentando a chance de óbito perinatal. Após a 33^a^ semana de gestação, recomenda-se realizar punções repetidas do cisto. Apresentamos um caso em que um dreno cístico-amniótico foi colocado e as punções foram evitadas.

**RELATO DE CASO:**

Um dreno cístico-amniótico foi colocado na 33^a^ semana de gestação devido a grande malfomação adenomatóide cística do pulmão associada a desvio de mediastino grave e polihidrâmnio. Apesar de o cateter ter sido identificado no local correto, o cisto voltou a crescer duas semanas após, repetindo o polihidrâmnio. Postulou-se que o cateter estava obstruído e optamos por colocar um novo cateter ao invés de realizar punções repetidas desse cisto. Foi observada regressão progressiva do volume do cisto, do polihidrâmnio e do desvio de mediastino. Na 38^a^ semana e meia, um menino de 3.310 g nasceu sem apresentar dificuldade respiratória, sendo submetido a toracotomia no 15^o^ dia de vida. Portanto, no presente estudo, discute-se sobre a possibilidade de colocação do dreno cístico-amniótico no lugar de realizar punções repetidas do cisto mesmo em idades gestacionais próximas do termo.

## INTRODUCTION

Congenital cystic adenomatoid malformation (CCAM) is a rare malformation that was first described by Chin and Tan in 1949. It consists of enlarged lung lobes with a tumorlike appearance, presenting multiple cysts or slit-like spaces. Histological examination reveals immature and disorganized pulmonary tissue with different degrees of lung malformation.^1^ Prenatal ultrasonographic findings are based on identifying pulmonary hyperechogenic images with microcystic or macrocystic lesions.^2^ In most cases with CCAM, the perinatal prognosis is favorable. However, a few cases with hydrops, polyhydramnios or large cysts have higher perinatal death rates.^2,3^ In cases of macrocystic CCAM with large cysts or associated with hydrops, cyst-amniotic shunt may reduce the cyst size and thus decrease the risk of perinatal death.^4^ This procedure is usually performed before the gestational age of 33 weeks. At a later stage, prenatal puncture of the adenomatoid cyst may be preferred.^4^ We report here a case of macrocystic CCAM in which a cyst-amniotic shunt was performed later, leading to reduction of the tumor mass and vaginal delivery without respiratory distress. The importance of this case report may lie in opening a discussion on fetal therapeutic options in such cases after the gestational age of 33 weeks.

## CASE REPORT

**Figure 1:**
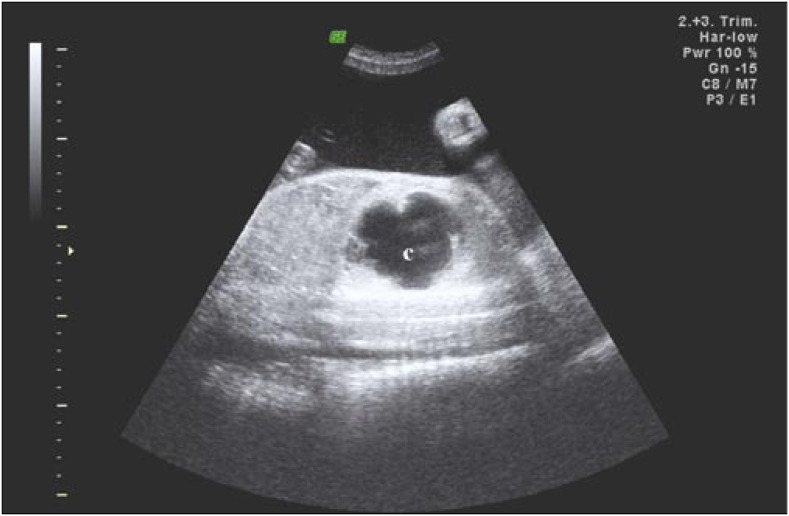
Sagittal section (two-dimensional ultrasound) through the left fetal thorax showing the hyperechogenic lung with a large cyst (c).

**Figure 2:**
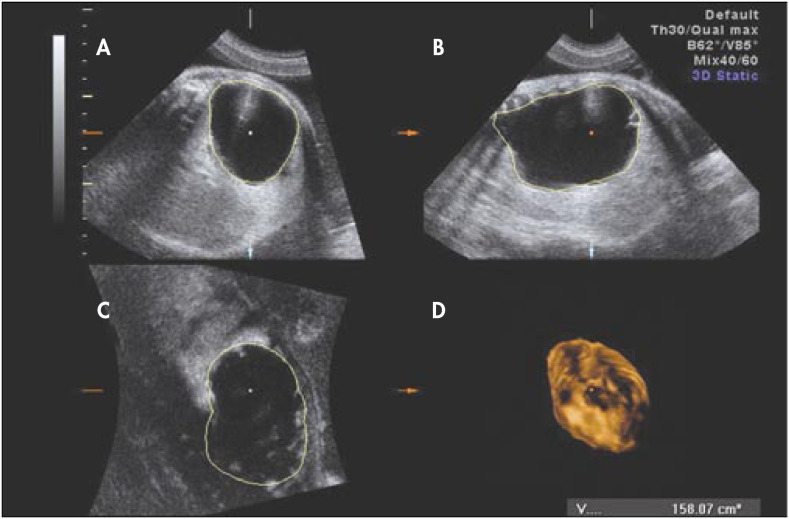
3D estimated volume of the adenomatoid cyst at 33 weeks: A) transverse section; B) sagittal section; C) coronal section; and D) rendered image.

**Figure 3:**
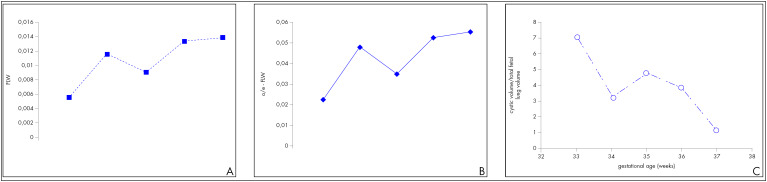
Evolution of cystic and lung volumes: A) estimated fetal lung/body weight ratio; B) observed/expected normal fetal lung volume ratio; and C) cyst/lung volume ratio.

We followed a 26-year-old woman in her fourth pregnancy (with one previous live birth and two previous abortions), who was referred to us at 30 weeks of gestation because of a prenatal diagnosis of CCAM. On ultrasound examination (Voluson 730-Expert, General Electric Medical System, Milwaukee, Wisconsin, United States), we diagnosed a CCAM in the left lung with moderate mediastinal shift, normal amniotic fluid volume and no other associated structural malformation. However, at 33 weeks, a great increase in size of the hyperechogenic lesion was observed, caused by a large cyst (Figure 1) associated with polyhydramnios (amniotic fluid index of 46) and preterm labor. The ratios of observed/expected fetal lung volume,^5^ ultrasonographic lung/body weight^6^ and cyst/lung volume were 0.22, 0.005 and 6.99 respectively (Figures 2 and 3).

The patient was hospitalized: tocolysis was performed using intravenous terbutaline for 12 hours, 2.5 liters of amniotic fluid were drained and a cyst-amniotic shunt (Harrison Fetal Bladder Stent Set, Cook Medical Inc., United States) was placed. Preterm labor was successfully inhibited, the fetal lung volume increased and the patient was discharged 24 hours after the procedure. However, two weeks later, the patient needed hospitalization again because of preterm labor associated with polyhydramnios (amniotic fluid index of 31) and increased size of the cystic lesion (cyst/lung volume ratio of 4.75). Since the catheter was seen to be still correctly in place, we hypothesized that a blockage was responsible for not allowing cystic drainage. On this occasion, we repeated the entire procedure, during which we drained two liters of amniotic fluid and placed a new cyst-amniotic catheter. The patient was discharged after 24 hours of preterm labor inhibition.

Regression of the cystic lesion was observed over the next two weeks, along with increasing fetal lung volume (Figure 3). The patient underwent spontaneous labor at 38.5 weeks with elective amniotomy at 5 cm of cervical dilatation. A 3,310 g male infant was delivered vaginally. Both catheters were taken off immediately after the delivery had progressed as far as the fetal shoulders. The newborn did not present any respiratory distress (Apgar scores of 9, 9 and 10 at the 1^st^, 3^rd^ and 5^th^ minutes respectively).

On the 15^th^ day of life, thoracotomy was performed and the cystic lesion was extirpated, while preserving the normal surrounding pulmonary tissue because of middle respiratory distress. The infant was discharged one week later, and no symptoms were presented up to the age of six months.

## DISCUSSION

Cyst-amniotic shunt has been usually performed in cases of macrocystic CCAM before the gestational age of 33 weeks.^4^ In the present case, we decided to place this shunt at 33 weeks because we observed that the cyst had developed recently and acutely and that it was responsible for severe mediastinal shift leading to polyhydramnios and preterm labor. Our first procedure gave a good result for one week. However, after one week, the cystic volume increased significantly, which again caused polyhydramnios and preterm labor. As we could see that one pigtail of the catheter was inside the cyst and the other extremity was located outside the fetal thorax, we speculated that a blockage inside the catheter was occurring. At this moment (35 weeks of gestation), we discussed the possibility of puncturing the adenomatoid cyst, in addition to draining the polyhydramnios. However, as this cyst had presented rapid evolution and increase (over a one-week period), we decided to place a new catheter. Our intention was to reduce the adenomatoid cystic volume and to allow delivery at full term. Another point was that this approach might avoid the need to perform perinatal puncturing of the cyst. Our concern was also to avoid the need to have a specialist in attendance during the delivery.

According to the literature, macrocystic CCAM can be successfully treated by placing a thoracoamniotic shunt, particularly in cases with severe mediastinal shift and/or hydrops.^4^ In all cases reported up to now that underwent a thoracoamniotic shunt, the procedure was performed before the gestational age of 33 weeks. It has been suggested that, at a later stage, a better therapeutic option might be prenatal puncturing of the adenomatoid cyst.^4^ However, multiple punctures might be needed, which might increase the risks of premature membrane rupture, infections and preterm labor.

Therefore, we believe that, even at a late gestational age, cyst-amniotic shunt placement may be better than performing repeated punctures of the cyst in cases of macrocystic CCAM. However, randomized studies comparing these two procedures at a late gestational age are still necessary. The objective of this case report was to stimulate further discussions on this issue.
